# Simple non-mydriatic retinal photography is feasible and demonstrates retinal microvascular dilation in Chronic Obstructive Pulmonary Disease (COPD)

**DOI:** 10.1371/journal.pone.0227175

**Published:** 2020-01-10

**Authors:** G. J. McKay, R. V. McCarter, R. E. Hogg, D. H. Higbee, M-P. K. Bajaj, D. R. Burrage, S. Ruickbie, E. H. Baker, P. W. Jones, J. W. Dodd

**Affiliations:** 1 Centre for Public Health, Queen’s University Belfast, Belfast, Northern Ireland; 2 Academic Respiratory Unit, Southmead Hospital, Bristol, United Kingdom; 3 Neuroscience Research Centre, Molecular and Clinical Sciences Research Institute, St George's, University of London, London, United Kingdom; 4 Clinical Pharmacology, Institute of Infection and Immunity Institute, St George’s, University of London, London, United Kingdom; University of Perugia, ITALY

## Abstract

**Background:**

Chronic Obstructive Pulmonary Disease (COPD) is associated with an increased risk of myocardial infarction and stroke but it remains unclear how to identify microvascular changes in this population.

**Objectives:**

We hypothesized that simple non-mydriatic retinal photography is feasible and can be used to assess microvascular damage in COPD.

**Methods:**

Novel Vascular Manifestations of COPD was a prospective study comparing smokers with and without COPD, matched for age. Non-mydriatic, retinal fundus photographs were assessed using semi-automated software.

**Results:**

Retinal images from 24 COPD and 22 control participants were compared. Cases were of similar age to controls (65.2 vs. 63.1 years, *p* = 0.38), had significantly lower Forced Expiratory Volume in one second (FEV_1)_ (53.4 vs 100.1% predicted; *p* < 0.001) and smoked more than controls (41.7 vs. 29.6 pack years; *p* = 0.04). COPD participants had wider mean arteriolar (155.6 ±15 uM vs. controls [142.2 ± 12 uM]; *p* = 0.002) and venular diameters (216.8 ±20.7 uM vs. [201.3± 19.1 uM]; *p* = 0.012). Differences in retinal vessel caliber were independent of confounders, odds ratios (OR) = 1.08 (95% confidence intervals [CI] = 1.02, 1.13; *p* = 0.007) and OR = 1.05 (CI = 1.01, 1.09; *p* = 0.011) per uM increase in arteriolar and venular diameter respectively. FEV_1_ remained significantly associated with retinal vessel dilatation r = -0.39 (p = 0.02).

**Conclusions:**

Non-mydriatic retinal imaging is easily facilitated. We found significant arteriole and venous dilation in COPD compared to age-matched smokers without COPD associated with lung function independent of standard cardiovascular risk factors. Retinal microvascular changes are known to be strongly associated with future vascular events and retinal photography offers potential to identify this risk.

**Trial registration:**

clinicaltrials.gov NCT02060292.

## Introduction

Chronic obstructive pulmonary disease (COPD) is the 4^th^ leading cause of death globally. In the UK, over one million people are diagnosed with COPD and an estimated further 2 million are affected [1]. COPD is frequently associated with co-morbidities such as cardiovascular disease, renal insufficiency, depression and osteoporosis, leading to the widely accepted view that COPD is a complex, multi-system disorder [2].

There is a high prevalence of traditional cardiovascular risk factors in patients with COPD including smoking, reduced physical activity and low socio-economic status. However, traditional cardiovascular risk factors do not appear to fully explain these findings, as numerous studies have shown airflow limitation to be independent of cardiovascular disease risk [3]. Patients with COPD have a significantly increased risk of cardiac events and stroke, particularly in early disease [4] and it is cardiovascular, not respiratory disease that is the leading cause of death and contributor to vascular comorbidities [5].

Smoking is a significant risk factor for COPD contributing to many of these associated complications with subsequent implications for the vasculature, blood pressure, and renal function, through oxidative stress, endothelial dysfunction, hypoxaemia and autonomic dysregulation [3, 6]. Nevertheless, mechanisms independent of smoking are also likely to be important in the disease process, manifesting in increased inflammation of the systemic circulation and contributing to the onset and progression of multiple comorbidities [7]. However, the extent of microvascular damage in COPD independent of smoking, remains unclear.

The Novel Vascular Manifestations of COPD (NOVASC) study provided detailed assessment of vascular pathology in COPD and age matched smokers without COPD [8]. Participants had brain and cardiac magnetic resonance imaging (MRI), in addition to measures of arterial stiffness and retinal photography. Arterial stiffness is a non-invasive measure of vascular function and a strong predictor of cardiovascular and cerebrovascular events leading to end-organ vascular damage through reduced vessel compliance, excessive pressure pulsatility resulting in vascular remodelling and impaired blood flow [9–10]. There is evidence of increased aortic stiffness in COPD independent of smoking, which is also associated with degree of airflow limitation and percentage emphysema on thoracic computed tomography [11].

Microvascular retinopathy is a strong predictor of both cardiac events and stroke in the general population and can be visualized through retinal fundus photography with high precision. Microvascular retinopathy is also associated with stroke independent of hypertension and may be useful for studying subclinical cerebrovascular disease [12]. In the large U.S. population based Multi Ethnic Study of Aging (MESA), arteriolar narrowing was independently associated with risk of stroke over 6 years follow up (hazard ratio [HR] = 3.01; 95% confidence intervals [CI]: 1.29–6.99) [13]. In the prospective Rotterdam study, retinal venular dilatation was independently associated with an increased risk of dementia over an 11 year follow up (HR = 1.31; 95% CI: 1.06–1.64) [14].

Retinal fundus photography offers an assessment of microvascular damage and offers an opportunity to improve our understanding of the mechanisms of microvascular pathology in COPD, identify patients with increased risk of future vascular events and a potential outcome measure for future intervention studies in COPD. To our knowledge, previous investigation of the retinal microvasculature in patients with COPD has been limited. A previous study reported significant associations between larger venular caliber and lower forced expiratory volume measured in one second (FEV_1_) and with lung density (p < 0.05) [15], while another investigation identified COPD as an independent determinant of microvascular retinopathy which correlates with disease severity and smoking status [16]. The factors that affect microvascular efficacy are complex, but the elevated risk of cardiac events that are associated with hypertensive/microvascular retinopathy and venular dilatation have been demonstrated [17].

Consequently, non-invasive measurement of the small vessel characteristics visualized in the retina provides a proxy for direct examination of the general microvasculature. We hypothesized that standard non-mydriatic retinal photography may provide evidence of microvascular damage in age-matched smokers with and without COPD. Therefore, we designed an observational case-controlled study and performed routine non-mydriatic retinal photography in patients with COPD as well as a control group of age and gender matched smokers. Our study achieved our aim by demonstrating that this form of retinal photography is feasible, and that there is significant retinal arteriolar and venular dilation in COPD compared to matched controls independent of potential confounders such as age, gender, smoking status, and cardiac risk factors such as MSBP, PWV and antihypertensive medications.

## Materials and methods

### Study design

This was an observational case-control study called the novel vascular manifestations of COPD (NOVASC) with patients recruited and all data collected over 12 months (January 2014- January 2015). The control group were age and gender matched without COPD. The NOVASC study sample size calculation was made in relation to the primary outcome of microstructural brain changes on MRI, for a conservatively estimated effect of d = 0.8, a sample of N = 30 compared against a sample of N = 25 will have in excess of 85% power (beta < 0.10) using alpha = 0.05, two-sided. Retinal analysis was informed by past empirical evidence and scientific reasoning and determined on feasibility and economic grounds and are comparable with past similar research [8]. Gradable retinal images of sufficient quality for vessel assessment were available in 46 participants. 1.0).

### Ethical approval

Ethical approval was given by the National Research Ethics Service Committee South West–Frenchay on 09/01/2014.

### Participants

The NoVasC study was designed to address the end organ impact of vascular pathology in COPD through direct comparison of COPD patients and smoking no disease controls. Recruitment of participants through GP practices in the Bristol area included individuals with COPD (aged > 40 and ≤ 85 years) diagnosed when the ratio of FEV_1_ to forced vital capacity (FVC) < 70%, without increase following bronchodilator treatment [18] and a smoking history of > 10 cigarette pack years. Study participants were not excluded for other diseases influencing retinal microvascular circulation such as diabetes or arterial hypertension. A gender and age matched control group was recruited from university non-healthcare staff and through local press advertisements. Controls were excluded if they had any self-reported memory difficulties or doctor-diagnosed respiratory disease. All participants provided written consent before inclusion into the study. The healthcare professional who was taking consent assessed capacity to understand, retain and recall information pertaining to consent. Additional exclusion criteria included where vessel caliber in the retinal images were ungradeable.

### Clinical features

Cognitive function was assessed using the Montreal Cognitive Assessment (MoCA) [19] other demographic and disease severity measures included a detailed medical history; arterialized earlobe capillary or radial artery blood gases whilst breathing room air; vital signs including oxygen saturation, heart rate, respiratory rate; health status questionnaire—COPD Assessment Test (CAT) [20]; and spirometry according to the American Thoracic Society/ European Respiratory Society guidelines [21]. Study visits took place at the Respiratory Research Unit, North Bristol Lung Centre.

### Retinal imaging and quantitative measurements of retinal microvasculature

**Fig 1:**
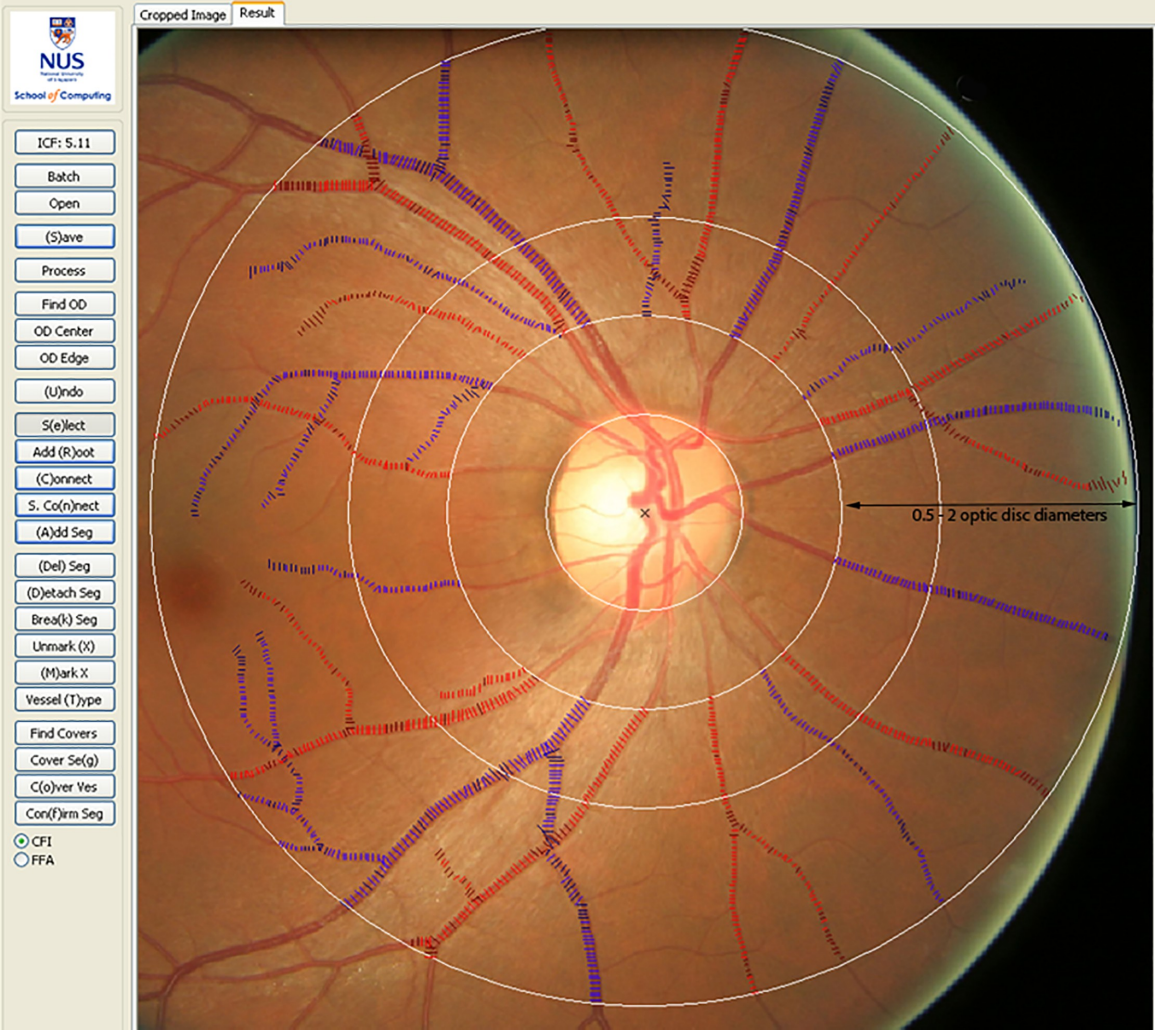
Retinal fundus image quantitatively assessed using the Singapore I Vessel Analyzer (SIVA) software. Arterioles are coloured red and venules in blue. The measurement region for retinal vascular parameters (caliber, fractal dimension, tortuosity and branching angle) was standardised from 0.5 to 2.0 optic disc diameters from the disc margin.

Participants underwent retinal color fundus photography. Retinal imaging took place at North Bristol medical illustration. A standard 45^0^ image was taken of each eye, with at least one image centered on the optic disc and the other on the macula. All retinal images were anonymized and examined. A trained grader who was proficient in the Singapore I Vessel Assessment (SIVA) at the Centre for Public Health (Queen’s University Belfast), masked to protect patient identity, used a standardized protocol to measure the retinal vessel caliber [22]. This was performed using the SIVA, a semi-automated computer assisted programme (version 3.0) to quantitatively measure retinal vascular parameters. The software automatically identifies the optic disc, centering a grid on it, calculating the retinal vascular parameters and identifying the vessel type. The measured area was standardized and defined within the region between 0.5 and 2.0 disc diameters away from the disc margin, and all visible vessels coursing through the specified zone were measured (Fig 1).

#### Retinal vascular calibre

The retinal arteriolar and venular calibers were summarized as central retinal arterial equivalent (CRAE) and central retinal venular equivalent (CRVE), respectively, according to the revised Knudtson-Parr-Hubbard formula [23]. The reproducibility of retinal vascular measurements was high, with a high intragrader reliability and an intraclass correlation coefficient previously reported calculated as 0.98 (CI: 0.97–0.98) for CRAE and 0.99 (CI: 0.99–0.99) for CRVE, respectively [22]. A high correlation between the right and left eyes in retinal vascular measurements has also been reported elsewhere [24]. Data from the right eye were used and when unavailable was replaced by left eye data.

#### Retinal vascular fractal dimension

Total, arteriolar, and venular fractal dimensions were determined from a skeletonized line tracing using the box counting method. These values represent a “global” summary measure of the whole branching pattern of the retinal vascular tree with larger values indicative of a more complex branching pattern [25].

#### Retinal vascular tortuosity

Retinal vascular tortuosity was estimated as the integral of the curvature square along the path of the vessel normalized by the total path length; this measure is dimensionless because it represents a ratio measure [26]. These estimates were summarized separately as retinal arteriolar and venular tortuosity [26]. Retinal vascular tortuosity reflects the straightness/waviness of the vessels; a smaller tortuosity value is indicative of a retinal vessel with a straighter path.

#### Retinal vascular branching angle

Retinal vascular branching angle was defined as the first angle subtended between two daughter vessels at each vascular bifurcation [27]. These estimates were summarized as retinal arteriolar branching angle and retinal venular branching angle, representing the average branching angle of arterioles and venules, respectively.

#### Other variables

Additional potential confounders were also considered within the conditional regression models: age; gender; smoking (pack years); mean systolic blood pressure (MSBP), PWV and anti-hypertensive medication.

### Statistical analysis

All statistical analyses were performed using IBM SPSS statistics version 21 (IBM Corp., Armonk, NY). An independent t test or χ2 test was used to compare the characteristics of COPD cases and controls in the study. Quantitative retinal vascular parameters were analyzed as continuous variables (and were standardized before entry into regression models to give estimates per standard deviation [SD] increase). Logistic regression models were used to analyze the association of retinal vascular parameters with COPD. Multiple logistic regression models were adjusted initially for age and gender (model 1), model 1 covariates and MSBP, smoking (measured in pack years)–model 2; and model 2 covariates with PWV and antihypertensive medication–model 3. Pearson’s correlation coefficient was used to calculate r value between lung function (FEV1) and retinal measures, adjusted for age, gender and smoking.

## Results

### Clinical features

Table 1 shows the summary characteristics of the COPD (n = 24) and control (n = 22) groups. The COPD participants had an average age of 65 years (range 50–78) and 54% (13) were male (Table 1). The control group (no COPD) was not significantly different in age to the COPD group with a mean age of 63 years (range 44–79). Cases had significantly lower (P < 0.001) mean FEV_1_ (%) (1.43; SD = 0.52) compared to controls (2.64; SD = 0.91) and FEV FVC (52.1 [12.7] versus 75.9 [9.9]). Cases were also more likely to be heavier smokers than controls (41.7 [20.8] pack years versus 29.6 [16.9]; P = 0.04). There were no significant differences between cases and controls in the other parameters measured.

**Table 1:** Summary characteristics of participants.

Characteristic	Cases, n = 24	Controls, n = 22	*P*
**Mean age, yrs (SD)**	65.2 (7.0)	63.1 (8.9)	0.38
**Mean MOCA**^**a**^ **(SD)**	25.9 (2.8)	27.0 (2.3)	0.17
**Male, n (%)**	13 (54)	10 (45)	0.56
**Mean SBP, mmHg (SD)**	143 (18)	143 (17)	0.90
**Smoking, mean pack years (SD)**	41.7 (20.8)	29.6 (16.9)	0.04
**Body Mass Index, kg/m**^**2**^ **(SD)**	28.3 (5.4)	27.0 (5.4)	0.41
**Mean FEV**_**1**_ ^**b**^ **(SD)**	1.43 (0.52)	2.64 (0.91)	<0.001
**Mean FEV**_**1**_ **FVC**^**c**^ **(SD)**	52.1 (12.7)	75.9 (9.9)	<0.001
**Mean eGFR**^**d**^**, ml/min/1.73m**^**2**^ **(SD)**	73.3 (17.6)	77.2 (10.1)	0.27
**Mean Albumin, mmol/l (SD)**	37.2 (2.7)	35.2 (11.2)	0.42
**Mean C reactive protein, mmol/l (SD)**	5.43 (5.36)	6.68 (11.9)	0.65
**Mean Pulse wave velocity, m/s (SD)**	10.6 (2.9)	11.0 (3.4)	0.67
**Antihypertensive medication, n (%)**	5 (21)	5 (23)	0.94

^a^ Montreal Cognitive Assessment

^b^ Forced expiratory volume in one second

^c^ Ratio of FEV_1_ to forced vital capacity

^d^ Estimated glomerular filtration rate

Gradable retinal images of sufficient quality for vessel assessment were available in 46 of the 51 participants. Table 2 shows the comparisons of retinal parameters between the COPD and control groups. Smokers with COPD had significantly wider mean arteriolar caliber (155.6 ±15 μM) versus no disease controls (142.2 ± 12 μM); *p* = 0.002 and mean venular diameters (216.8 ±20.7 μM vs. 201.3± 19.1 μM): *p* = 0.012. No significant variations in arteriolar or venular fractal dimension, branching angles or tortuosity were detected in both unadjusted and adjusted analyses between groups (P > 0.05).

**Table 2:** Comparisons of retinal vascular parameters between COPD cases and controls.

Parameter	COPD^a^ cases, n = 24	Controls, n = 22	P^b^
	Mean (SD^c^)	Mean (SD)	
**Caliber**			
** Central retinal arteriolar equivalent, μm**	155.6 (15.0)	142.2 (12.0)	0.002
** Central retinal venular equivalent, μm**	216.8 (20.7)	201.3 (19.1)	0.012
**Fractals**			
**Total fractal dimension**	1.366 (0.043)	1.365 (0.064)	0.921
** Arteriolar fractal dimension**	1.164 (0.052)	1.173 (0.066)	0.604
** Venular fractal dimension**	1.149 (0.049)	1.138 (0.067)	0.531
**Tortuosity**			
** Arteriolar tortuosity (x10**^**4**^**)**	0.697 (0.210)	0.639 (0.132)	0.275
** Venular tortuosity (x10**^**4**^**)**	0.709 (0.220)	0.644 (0.164)	0.264
**Bifurcations**			
** Arteriolar branching angle,** °	79.44(12.89)	77.62 (19.00)	0.703
** Venular branching angle,** °	78.22 (12.86)	75.33 (27.04)	0.641

^a^ Chronic obstructive pulmonary disease

^b^ P values were calculated by independent sample *t* test.

^c^ Standard deviation

°degree

In multivariate logistic regression, persons with larger arteriolar (OR per SD increase, 4.02 [CI: 1.54–10.5]) and venular caliber (OR per SD increase, 2.91 [CI: 1.16–7.33]) were more likely to have COPD following adjustment for age, gender, smoking status (pack years), PWV, MSBP and antihypertensive medication (Table 3, model 3). When controlling for age, gender, BP, smoking (pack years), the association between FEV_1_% predicted (combined arteriolar and venular) retinal vessel dilation was r -0.389 (p = 0.02).

**Table 3:** Associations between COPD and retinal vascular parameters.

Parameter	Model 1	Model 2	Model 3
	OR^a^ (95% CI^c^); P^b^	OR (95% CI); P	OR (95% CI); P
**Caliber**			
** Central retinal arteriolar equivalent per SD increase**	2.99 (1.36–6.60); 0.006	3.39 (1.41–8.17); 0.007	4.02 (1.54–10.5); 0.005
** Central retinal venular equivalent per SD increase**	2.89 (1.27–6.57); 0.012	3.01 (1.26–7.23); 0.013	2.91 (1.16–7.33); 0.023
**Fractals**			
**Total fractal dimension per SD increase**	1.10 (0.59–2.02); 0.768	1.12 (0.58–2.17); 0.734	1.17 (0.57–2.37); 0.670
** Arteriolar fractal dimension per SD increase**	0.89 (0.48–1.65); 0.713	0.98 (0.50–1.90); 0.940	1.04 (0.52–2.11); 0.908
** Venular fractal dimension per SD increase**	1.28 (0.69–2.36); 0.437	1.14 (0.59–2.23); 0.692	1.13 (0.56–2.26); 0.729
**Tortuosity**			
** Arteriolar tortuosity per SD increase**	1.40 (0.73–2.67); 0.307	1.83 (0.85–3.94); 0.120	2.13 (0.93–4.91); 0.075
** Venular tortuosity per SD increase**	1.42 (0.72–2.78); 0.314	1.55 (0.76–3.17); 0.229	1.51 (0.72–3.18); 0.275
**Bifurcation**			
** Arteriolar branching angle per SD increase**	1.14 (0.62–2.11); 0.666	0.94 (0.47–1.87); 0.863	0.99 (0.49–2.03); 0.990
** Venular branching angle per SD increase**	1.13 (0.61–2.08); 0.704	0.97 (0.51–1.83); 0.921	1.02 (0.53–1.96); 0.955

Model 1 was adjusted for age and gender. Model 2 was adjusted for age, gender, mean systolic blood pressure, smoking status (pack years). Model 3 was adjusted for model 2 covariates, pulse wave velocity and antihypertensive medications.

^a^ Odds ratio

^c^ Confidence intervals

^b^ P values

## Discussion

This study has demonstrated that routine non-mydriatic retinal photography is feasible in COPD and that there is significant retinal arteriolar and venular dilation in COPD compared to matched controls independent of potential confounders such as age, gender, smoking status, and cardiac risk factors such as MSBP, PWV and antihypertensive medications (P < 0.05). Microvascular dilatation has been reported previously in smokers, both in healthy people without overt cardiovascular or metabolic disease [28] and in persons with COPD [16]. It is also consistent with the MESA lung epidemiology study which reported associations between larger venular (not arteriolar) caliber and lower FEV_1_ and lung density [15]. The significance of smoking is complex, often manifesting in long-term consequences for microvascular systems. It contributes to elevated cardiovascular and renovascular risk with venular dilatation resultant from toxin-induced endothelial damage, moderated vasoactive intermediaries, tissue hypoxaemia and autonomic dysregulation [3, 6], suggesting venular widening may lead to lower oxygen saturation [29]. Retinal vascular caliber may revert to normal after a specific period of time of sustained smoking cessation [30]. As such, retinal venular caliber could become a useful tool to monitor and estimate COPD risk.

The correlation of microvascular abnormalities in COPD patients has been recently reviewed by Vaes and colleagues in 2018 [31]. Peripapillary choroidal thickness in COPD patients has been examined in three studies [32–34]. Ozcimen and colleagues found that the COPD patients had significantly thinner peripapillary choroid in the inferior segments compared to other peripapillary choroid segments and thinner than the controls [32]. Choroidal thinning in COPD may be due to vascular endothelial dysfunction and hypoxia, which can make the choroid more susceptible to diseases in the retina [32], and systemic microvascular disorders [35]. However, there was no difference in the mean peripapillary and subfoveal choroidal thickness in COPD compared to controls [32].

The strengths of this study are in its comprehensive assessment of vascular pathology in a well characterized population of patients with COPD and the use of a healthy smoking control group to help determine the contribution of COPD specific effect on vascular damage. We used a readily available direct measure of microvascular damage assessed with validated software. The images were collected in a ‘real world’ clinical environment without specialist training or the need for mydriatic eye drops. However in this detailed mechanistic study, participant numbers were small limiting definitive conclusions to be made regarding the presence or absence of association between retinal vascular features and disease and demographic measures. In addition, there may be residual confounding factors not measured in our sample or considered in our data. Any causal and temporal relationships between vessel caliber and COPD cannot be determined due to the cross-sectional nature of our study, which prevents the inference of any causal relationship. We therefore restrict our conclusions to those associations that remain significant after comprehensively controlling for confounders. For the results that indicated no significant association, type II error may occur as the study power was limited by the number of events and the number of potential confounders included within the statistical models. There are also some conditions that may potentially influence the microvasculature of the retina. Specifically, the narrowing of the retinal arterioles are independent predictors of incident diabetes mellitus, coronary heart disease and hypertension, while the widening of retinal venules has been associated with incident cardiovascular disease [36–39].

In summary, our data provides evidence of significant retinal arteriolar and venous dilatation in COPD compared to age-matched smokers without COPD. These differences are not fully explained by standard cardiovascular risk factors, including smoking, and are associated with lung function. The ability of venular dilatation to predict cardiac events and stroke have been demonstrated in the non-COPD population [17]. Retinal microvascular measures can be feasibly collected in clinic and offer the opportunity to identify future vascular events associated with COPD. Further longitudinal studies would be valuable in establishing if this is the case and evaluation of the sensitivity and specificity of the microvascular changes detected. The integrity of the microvasculature is extremely complex and a variety of risk factors influence the retinal arteriolar and venular bed, which may provide differential prognostic significance for both systemic and chronic diseases.

## Supporting information

S1 Checklist TREND statement checklist. (PDF)

S1 Protocol NOVASC protocol. (DOC)

S1 Flow Chart (TIFF)

## List of abbreviations

CRAE: central retinal arterial equivalent
CRVE: central retinal venular equivalent
COPD: chronic obstructive pulmonary disease
CI: confidence intervals
ECG: electrocardiogram
FEV_1_: forced expiratory volume measured in one second
FVC: forced vital capacity
HR: hazard ratio
MRI: magnetic resonance imaging
MSBP: mean systolic blood pressure
NOVASC: The Novel Vascular Manifestations of COPD
PWV: pulse wave velocity
SD: standard deviation
