# Supplementary material for: Simple non-mydriatic retinal photography is feasible and demonstrates retinal microvascular dilation in Chronic Obstructive Pulmonary Disease (COPD)
Source: PLoS One. 2020 Jan 10;15(1):e0227175. doi: 10.1371/journal.pone.0227175 (PMC6953864; doi:10.1371/journal.pone.0227175)
Supplement: S1 Protocol — (DOC) [file pone.0227175.s002.doc]

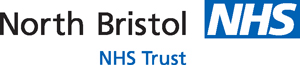

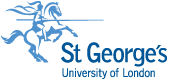


**N.O.V.A.S.C**

**N**ovel **Vasc**ular Manifestations of **COPD**

PROTOCOL

SPONSOR: North Bristol NHS Trust

FUNDERS: Academy of Medical Sciences / NIHR.

STUDY COORDINATION CENTRE: North Bristol NHS Trust

Chief investigator: Dr. James Dodd

Study Protocol Version: 1.0 (4^th^ November 2013)

REC Ref:

R&I Number: 3251

**Authorised by:**

Name: Dr. J W. Dodd

Role: Chief Investigator

Date: **SIGNATURE**

This protocol describes the Novel Vascular Manifestations of COPD study and provides information about procedures for entering participants. Every care was taken in its drafting, but corrections or amendments may be necessary. These will be circulated to investigators in the study. Problems relating to this study should be referred, in the first instance, to the Chief Investigator.

This study will adhere to the principles outlined in the NHS Research Governance Framework for Health and Social Care (2nd edition). It will be conducted in compliance with the protocol, the Data Protection Act and other regulatory requirements as appropriate.

**Study Management Group**

**Chief Investigator**:

Dr James W Dodd

Academic Clinical Lecturer Respiratory Medicine

Academic Respiratory Unit

Floor 2, Learning and Research Building

Southmead Hospital

Bristol BS10 5NB

[james.dodd@bris.ac.uk](mailto:james.dodd@bris.ac.uk)

Tel: +44 (0)117 3238725

**Co-investigators**:

Prof Emma Baker

Professor of Clinical Pharmacology & Consultant Physician Respiratory Medicine

Division of Biomedical Sciences

St George’s University of London

Cranmer Terrace, Tooting

London, SW19 0RE

[ebaker@sgul.ac.uk](mailto:ebaker@sgul.ac.uk)

Tel: +44 (0)20 8725 5383

Prof Paul W Jones

Professor of Respiratory Medicine

Head of Division of Clinical Science

St George's, University of London

Cranmer Terrace. Tooting

London SW17 0RE

[pjones@sgul.ac.uk](mailto:pjones@sgul.ac.uk)

Tel +44 (0) 20 8725 5371

Fax +44 (0) 20 8725 5955

**Statistician**:

Dr Paul White

Director of Applied Statistics Group

University of West of England

[p.white@uwe.ac.uk](mailto:p.white@uwe.ac.uk)

0117 32 83777

**Study Coordination Centre**

For general queries, supply of study documentation, and collection of data, please contact:

Deborah Warbrick

Senior Research Nurse

Respiratory Research Unit

Southmead Hospital

Bristol. BS10 5NB

Tel: 0117 323 5838

**Clinical Queries**

Clinical queries should be directed to the chief investigator who will direct the query to the appropriate person

**Sponsor**

North Bristol NHS Trust is the research sponsor for this trial. For further information regarding the sponsorship conditions, please contact: Helen Lewis

**Funder** Academy of Medical Sciences / NIHR

**TABLE OF CONTENTS**

**1. INTRODUCTION**

1.1 Background & Rationale

**2. STUDY OBJECTIVES**

**3. STUDY DESIGN**

3.1 Study outcome measures

**4. PARTICIPANT ENTRY**

4.1 Pre-registration evaluations

4.2 Inclusion criteria

4.3 Exclusion criteria

4.4 Withdrawal criteria

**5. ADVERSE EVENTS**

5.1 Definitions

5.2 Reporting procedures

**6. ASSESSMENT AND FOLLOW-UP**

**7. STATISTICS AND DATA ANALYSIS**

**8. REGULATORY ISSUES**

8.1 Ethics approval

8.2 Consent

8.3 Confidentiality

8.4 Indemnity

8.5 Sponsor

8.6 Funding

8.7 Audits and inspections

**9. STUDY MANAGEMENT**

**10. PUBLICATION POLICY**

**11. REFERENCES**

1. **INTRODUCTION**

Chronic obstructive pulmonary disease (COPD) is the 4th leading cause of death globally. In the UK, over a million people are diagnosed with COPD and an estimated further 2 million are affected . COPD is frequently associated with co-morbidities such as cardiovascular disease, renal insufficiency, depression and osteoporosis, leading to the widely accepted view that COPD is a complex multi-system disorder . Funding bodies including MRC have highlighted the need to determine new therapeutic strategies, including the extra pulmonary manifestations of COPD.

**1.1 BACKGROUND & RATIONALE**

Early studies of cognitive performance in COPD tended to focus on factors associated with functional neuronal impairment, namely hypoxaemia, but cognitive impairment is present in the absence of hypoxaemia and explains only a small proportion of the variance in cognitive ability in those patients with COPD who are hypoxaemic . Two recent studies suggest that it is occult cerebrovascular damage that plays a key role in brain damage and dysfunction in COPD. In the first study, we examined brain pathology in patients with stable COPD using MRI techniques to provide sensitive measures of white matter microstructure and grey functional activation. This showed widespread damage to white matter integrity and disturbance in functional activation of grey matter consistent with microvascular pathology . The second study, a European community cohort, suggests that obstructive lung disease is related to an increased risk of the developing of cerebral microbleeds (a marker of cumulative small vessel damage) .

Other neuroimaging studies relevant to brain pathology in COPD are mixed. Smoking has been shown to be associated with cerebral atrophy and reduced frontal grey matter density . However large population studies suggest that factors other than smoking are involved since impaired lung function is associated with cerebral white matter lesions independent of smoking and in non-smokers . Studies in well characterised patients with COPD are lacking, one study of male patients with “chronic respiratory disease” found them to have increased white matter lesions and cerebral atrophy , in contrast a small study comparing COPD patients with and without hypoxaemia showed not difference in brain volume or white matter lesions . Therefore questions remain about the precise contribution of smoking, impact on cognitive function and underlying pathophysiological mechanisms of brain pathology in COPD .

There is a high prevalence of traditional cardiovascular risk factors in patients with COPD including smoking, reduced physical activity and low socio-economic status. However, numerous studies have shown that airflow limitation is an independent risk factor for cardiovascular disease . Patients with COPD are at a significantly increased risk of cardiac events and stroke particularly in early disease and it is cardiovascular, not respiratory disease that is the leading cause of death . This evidence points to a potential 'COPD specific effect' that contributes to the increased vascular comorbidities.

Arterial stiffness is a non-invasive measure of vascular function and accurately predicts cardiovascular and cerebrovascular events. In addition, arterial stiffness is thought to contribute directly to end-organ vascular damage through reduced vessel compliance, excessive pressure pulsatility resulting in vascular remodelling and impaired blood flow . There is evidence of increased aortic stiffness in COPD, independent of smoking, which also relates to degree of airflow limitation and percent emphysema on thoracic CT . It has been suggested that arterial stiffness in COPD may be due to increased susceptibility to degradation of connective tissue or accelerated aging. These factors are also implicated in the development of emphysema suggesting a potential shared pathophysiology between pulmonary and vascular disease in COPD .

A recent comprehensive review of vascular disease in COPD presents several plausible mechanisms including systemic inflammation (IL-6, CRP), oxidative stress (through activation of matrix metalloproteinases), physiological stress (hypoxia, sympathetic nervous system activation), arterial stiffness, accelerated aging and protease/antiprotease inbalance. It is argued that many of these pathways are abnormal in COPD, independently predict cardiovascular disease, e.g. through atherosclerosis formation or vascular damage and also show direct pathophysiological links to the development of emphysema .

Cognitive impairment is a known consequence of cerebral small-vessel disease. Moderate to severe cognitive impairment has been shown in up to 60% of certain individuals with COPD , which may predict death and is likely to profoundly influence an individual’s ability to manage their disease . Therefore understanding the relationship between cerebrovascular disease and cognitive impairment in COPD is a research priority.

In addition to cerebral small vessel damage and cognitive dysfunction, other organs such as the heart, kidneys, and retinal are likely to be susceptible to small-vessel damage in COPD. Several large population studies have shown that COPD is a significant independent risk factor for myocardial infarction, with the effect most marked in early, mild disease and that people diagnosed and treated COPD are at increased risk for hospitalizations and deaths due to cardiovascular diseases . Acute exacerbations of COPD are inflammatory episodes associated with platelet activation, troponin rise and subsequent increased cardiovascular events . Left ventricular mass predicts heart failure and cardiovascular mortality and has been found to be increased in COPD and associated with pulmonary hyperinflation . A small exploratory study suggests that occult myocardial infarction may be responsible for delayed cardiac MR enhancement in COPD , another suggests right ventricular hypertrophy on cardiac MR is and early sign of RV overload . Finally percentage of emphysema and lung function have been found to be inversely associated with left ventricular filling, reduced stroke volume and cardiac output with preservation of ejection fraction .

Chronic renal failure is highly prevalent in elderly patients with COPD and associated with systemic inflammation but is a relatively understudied co-morbidity. Micro-albuminuria has been shown to be an independent predictor of future cardiovascular events and a marker of vascular endothelial dysfunction . To our knowledge, retinal vessels have not been extensively investigated in patients with COPD. Retinal photography is a sensitive and precise method of assessing retinal microvascular signs and is thought to be a potential marker for concomitant cerebral microangiopathy. Population based studies indicate that retinal microvascular changes, when defined from photographs, are associated with stroke independent of hypertension may be useful for studying subclinical cerebrovascular disease .

We propose to compare non-invasive MR brain imaging of white matter microstructure (diffusion tensor), cerebral perfusion (arterial spin labelling) and accumulated cerebral small vessel disease (cerebral microbleeds), in COPD patients to smokers without COPD.

This will also be the first study to explore mechanisms of cerebral small vessel disease in COPD by looking for associations between arterial stiffness, end organ vascular damage and cognitive function.

This research will provide essential data required to fund longitudinal and intervention studies designed to prevent vascular manifestations and the associated morbidity and mortality in COPD.

1. **STUDY HYPOTHESIS & OBJECTIVES**

Hypothesis:

1. COPD is associated with cerebral small vessel disease independent of smoking.
2. Cerebral small vessel disease in COPD is associated with aortic stiffness, cognitive function, cardiac, retinal & renal microvascular damage and systemic inflammation.

Objectives:

1. Test whether MRI measures of cerebral small vessel disease in COPD is independent of cigarette smoking by comparing to age and smoking history matched controls.
2. Explore possible mechanistic relationships between MR measures of cerebral small vessel disease and perfusion in COPD with pulse wave velocity and markers of systemic inflammation.
3. Determine if MRI measures of cerebral small vessel disease and perfusion are associated with measures of microvascular damage in other susceptible organs (cardiac, retina and renal) in COPD.
4. Determine if MR measures of cerebral small vessel disease and perfusion in COPD are associated with cognitive function.
5. **STUDY DESIGN**

Prospective recruitment, to an observational case control study with cross-sectional analysis designed to compare 30 well-characterised COPD cases with 25 healthy smoker controls.

The broad timetable for the stages of the research

Preparation August 2013 - Dec 2013 (4 months)

Recruitment & Data Collection Jan 2014 - Jan 2015 (12 months)

Interpreting & analysing findings Feb 2015 - March 2015 (1 month)

Preparing the final report. April 2015 - May 2015 (1 month)

*Study visits will take place at the Respiratory Research Unit, North Bristol Lung Centre.

*Retinal imaging which will take place at north Bristol medical illustration and MRI which will take place at the Clinical Research imaging Centre (CRIC) Bristol 3T MRI scanner.

Recruitment will be from patients attending clinic at local University Teaching Hospitals. An age-matched control group will be recruited from the Bristol Primary care Research Network North Hub, local community either through local press advertisements or University non-healthcare staff. Controls will be excluded if they have any respiratory or memory difficulties.

All Participants will be invited for an MRI, retinal photography and 2 further study visits to undergo non-invasive assessment of aortic stiffness and collect the measures outlined below. The total duration of the study should be ~ 2½ hours.

**3.1 STUDY OUTCOME MEASURES**

**Primary outcome**

MRI: White Matter microstructural damage (Fractional Anisotropy)

**Secondary Outcomes**

1. Aortic Stiffness: Pulse Wave Velocity
2. MRI: Number of cerebral microbleeds (CMB)
3. MRI: Cerebral perfusion (Arterial Spin Labelling – ASL)
4. Cognitive Function (Montreal Cognitive Assessment – MoCA)
5. Systemic inflammation, (C reactive protein, Interleukin 6, Fibrinogen)
6. Cardiac MR (RV, LV mass, cardiac output)
7. Micro-albuminuria
8. Retinal arteriolar narrowing (arteriole to venule ratio)
9. Forced Expiratory Volume in 1 second (FEV_1_ % predicted)
10. Health Status
11. Arterial partial pressure of oxygen

**Measures**

**3Tesla MRI:**

University of Bristol Clinical Research Imaging Centre (CRIC) [www.bristol.ac.uk/cricbristol](http://www.bristol.ac.uk/cricbristol)

*Summary Brain Imaging Protocol ~ 45min***.**

1. Anatomy / Volume
2. T1 FLAIR (Evidence of burden and pattern of vascular disease)
3. Diffusion Tensor Imaging (micro structure / evidence of recent ischaemia)
4. Resting Functional Imaging (marker of cerebral activity)
5. T2 Susceptibility weighted imaging (cerebral micro-bleeds)
6. Arterial Spin Labelling (measure of regional tissue perfusion

*Summary Cardiac Imaging Protocol ~ 20min*

1. Non-invasive imaging including fast gradient-echo cine images of the left and right ventricles.

**Aortic Stiffness :**

Carotid-femoral pulse wave velocity, augmentation index and central pressures.

Subjects will be studied after an overnight fast and 6 hours after abstinence from caffeine, tobacco, and inhaled short-acting β2 agonists. Therefore this assessment cannot be made on the same day as when consent is taken, but on a subsequent study visit. All tests are to be performed after 10 minutes of supine rest. After peripheral blood pressure is measured, radial artery waveforms are recorded with a high-fidelity micromanometer (Millar Instruments, Houston, TX). Pulse wave analysis (Sphygmocor; AtCor Medical, Sydney, Australia) will be used to generate a corresponding central waveform, using a validated transfer function . With the integral software, Augmentation Index will be calculated as the difference between the second and first systolic peaks as a percentage of pulse pressure. Aortic Pulse Wave Velocity (PWV) will be measured with the same device by sequentially recording ECG-gated carotid and femoral artery waveforms. Wave transit time will be calculated by the system software, using the R wave of a simultaneously recorded ECG as a reference frame. Aortic PWV was determined by dividing the distance between the two recording sites by the wave transit time .

**Retinal Photography:**

Non-mydratic, macular focused retinal photography will be collected by qualified technicians with the study site department of medical illustration. Generalised retinal arteriolar narrowing will be estimated by measuring retinal vessel diameters from photographs by use of validated imaging software. Reproducibility from photographs is excellent (kappa 0.80 – 0.99) (22).

**Renal Function:**

Serum, estimated Glomerular Filtration Rate (eGFR). Urine dipstick for microalbuminuria. Urine albumin creatinine ratio.

**Cognition Assessment:**

Montreal Cognitive Assessment (MoCA) is a well validated clinical tool used to assess all major cognitive domains. It will be administered by an appropriately trained study co-ordinator as per developer protocol and completed during the study visit.

**Other demographic and disease severity measures:**

1) Detailed medical history

2) Arterialised earlobe capillary or radial artery blood gases whilst breathing room air

3) Blood pressure in resting state x 3

4) Vital signs including Oxygen saturation, Heart Rate, Respiratory rate

5) Health Status Questionnaire - COPD Assessment Test (CAT) .

6) Mood Questionnaire - Hospital Anxiety & Depression (HAD) questionnaire .

9) Spirometry: ATS/ ERS guidelines

10) Snellen chart (visual acuity)

11) Blood tests: Full Blood Count, Urea and Electrolytes, Fasting Glucose and Lipids

**Management of Biological Samples:**

Routine serum samples will be collected in EDTA and SSC vacutainers during study visit 2 prior to vascular ultrasound. Study investigators will process clinical samples through the study site general pathology services. Immediately after being centrifuged, anonymised samples will be storage in a dedicated 80 degrees centigrade freezer at North Bristol NHS. This location has restricted access to trial research staff and approved lab staff only. The freezers are protected by a failsafe mechanism which ensures sample integrity by alerting staff in the event of failure.

**4. PARTICIPANT ENTRY**

Recruitment will be from inpatients and outpatient respiratory clinics at University Teaching Hospitals. An age-matched control group will be recruited from the local community either through local press advertisements or University non-healthcare staff. Controls will be excluded if they have any physician-diagnosed respiratory disease or self-reported memory difficulties.

**4.2 INCLUSION CRITERIA**

**Subjects for enrolment in the study must meet the following criteria:**

- - 1. Subjects must give their signed and dated written informed consent
    2. Subjects must be aged >40 and ≤ 85

**COPD Cohort:**

Subjects with a FEV/FVC <70%

Subjects with a smoking history > 10 pack years. Ex-smokers.

**Healthy Ex-smoker Controls**:

Subjects must have a FEV1/FVC >70%;

Patient and control group will be matched for smoking history and age.

**4.3 EXCLUSION CRITERIA**

Subjects meeting any of the following criteria must not be enrolled in the study:

Resting oxygen saturations <92% on room air.

Long term oxygen therapy

Recent exacerbation of COPD (4 weeks)

Ischaemic heart disease

Cerebrovascular disease

Uncontrolled hypertension

Diabetes mellitus

Hepatic failure

Neurological disease

Non-cured tumours

Obstructive sleep apnoea

Current of past alcohol/drug abuse

Known history of dementia

Visual or hearing impairment that precludes neuropsychological assessment

Neuropsychological tests undertaken outside the study

Pregnant women or women who are lactating

Known alpha 1 anti-trypsin deficiency as a cause of their COPD

History of psychiatric disorders, or other conditions that will impact on the validity of their consent or interfere with compliance to perform study procedures.

Contra indication to MRI scanning

**5. ADVERSE EVENTS**

**5.1 DEFINITIONS**

**Adverse Event (AE):** any untoward medical occurrence in a patient or clinical study subject.

**Serious Adverse Event (SAE):** any untoward and unexpected medical occurrence or effect that:

- **Results in death**
- **Is life-threatening** – *refers to an event in which the subject was at risk of death at the time of the event; it does not refer to an event which hypothetically might have caused death if it were more severe*
- **Requires hospitalisation, or prolongation of existing inpatients’ hospitalisation**
- **Results in persistent or significant disability or incapacity**
- **Is a congenital anomaly or birth defect**

Medical judgement should be exercised in deciding whether an AE is serious in other situations. Important AEs that are not immediately life-threatening or do not result in death or hospitalisation but may jeopardise the subject or may require intervention to prevent one of the other outcomes listed in the definition above, should also be considered serious.

**5.3 REPORTING PROCEDURES**

All adverse events should be reported. Depending on the nature of the event the reporting procedures below should be followed. Any questions concerning adverse event reporting should be directed to the Chief Investigator in the first instance. The case report period is from time of consent until the last trial administered procedure at visit 4.

**5.3.1 Non serious AEs**

All such events, whether expected or not, will be recorded in the Case Report File (CRF)

**5.3.2 Serious AEs**

In the case of a SAE occurring, an SAE form should be completed and faxed to the trial coordination centre and to the sponsor within 24 hours. hospitalisations for elective treatment of a pre‐existing condition do not need reporting as SAEs.

All SAEs should be reported to the <name of REC> where in the opinion of the Chief Investigator, the event was:

- ‘related’, ie resulted from the administration of any of the research procedures; and
- ‘unexpected’, ie an event that is not listed in the protocol as an expected occurrence

Reports of related and unexpected SAEs should be submitted within 15 days of the Chief Investigator becoming aware of the event, using the NRES SAE form for non-IMP studies. The Chief Investigator must also notify the Sponsor of all SAEs.

Local investigators should report any SAEs as required by their Local Research Ethics Committee, Sponsor and/or Research & Development Office.

**SAE forms should be submitted by**

**Fax: 0117 323 8691 attention James Dodd/Deborah Warbrick**

**Please send SAE forms to:**

**N.O.V.A.S.C study**

**Respiratory Research Unit**

**Southmead Hospital**

**Bristol**

**BS10 5NB**

**Tel: 0117 323 5838 (Mon to Fri 09.00 – 17.00)**

**6. ASSESSMENT AND FOLLOW-UP**

Patients will have 4 visits. Attempts will be made to co-ordinate the study visits to suit the patient and combine them if possible / desired by the participant.

- Visit 1: Consent and collection of medical history, measures of disease severity and completion of questionnaires - much of this information will be similar to that collected in routine clinical care of patients with COPD. Lung function, health related quality of life and mood questionnaires and cognitive assessment.
- Visit 2: Non-invasive assessment of aortic stiffness, arterial blood gas and venous bloods.
- Visit 3: MRI scanning. The brain scanning protocol will last approximately 45 minutes following by a 10 minute comfort break and a further 30 minutes of cardiac scanning; resulting in a total study visit of approximately 1½ hour.
- Study Visit 4. Retinal photography. This is a short non-invasive procedure that lasts a few minutes. We anticipate the entire visit will last less than 30 minutes.

Study endpoint will be when last patient has completed last visit.

Patient withdrawal: If a patient decides to withdraw their consent during the study, then the study investigator will complete the withdrawal of consent form on the CRF. Subject to ethical approval, data already collected in relation to the participant may be retained and used for the purposes for which consent has already been given, provided they are effectively anonymised and no longer identifiable to the research team or any other persons to whom access will be given.

**7. STATISTICS AND DATA ANALYSIS**

The proposed study analysis is, informed by past empirical evidence and scientific reasoning. It is designed to investigate potentially important differences in both brain pathology, and vascular function, between COPD and Smoker groups and to investigate associations between brain pathology and vascular function. Standard statistical techniques (independent sample t-test or nonparametric Mann Whitney test as appropriate and similar) will be used. In these respects a sample size of N = 55 will be taken with N = 30 sampled from the COPD population and with a comparator group of N = 25 from a smoker population. These sample sizes have been determined on feasibility and economic grounds and are comparable with past similar research . These studies showed very large effect sizes for a two-group comparison (Cohen’s d in excess of 1.0). For a conservatively estimated effect of d = 0.8, a sample of N = 30 compared against a sample of N = 25 will have in excess of 85% power (beta < 0.10) using alpha =0.05, two-sided.

- Data and all appropriate documentation will be stored for a minimum of 10 years after the completion of the study, including the follow-up period.

**8. REGULATORY ISSUES**

**8.1 ETHICS APPROVAL**

The Chief Investigator has obtained approval from the xxx Research Ethics Committee. The study must be submitted for Site Specific Assessment (SSA) at each participating NHS Trust. The Chief Investigator will require a copy of the Trust R&D approval letter before accepting participants into the study. The study will be conducted in accordance with the recommendations for physicians involved in research on human subjects adopted by the 18th World Medical Assembly, Helsinki 1964 and later revisions.

**8.2 CONSENT**

Consent to enter the study must be sought from each participant only after a full explanation has been given, an information leaflet offered and time allowed for consideration. Signed participant consent should be obtained. The right of the participant to refuse to participate without giving reasons must be respected. After the participant has entered the study the clinician remains free to give alternative treatment to that specified in the protocol at any stage if he/she feels it is in the participant’s best interest, but the reasons for doing so should be recorded. In these cases the participants remain within the study for the purposes of follow-up and data analysis. All participants are free to withdraw at any time from the protocol treatment without giving reasons and without prejudicing further treatment.

**8.3 CONFIDENTIALITY**

The Chief Investigator will preserve the confidentiality of participants taking part in the study and is registered under the Data Protection Act.

Following consent, participants will be assigned a unique trial number, this will be used on CRFs, other trial documents and the electronic database. The documents and database will also use their initials (of first and last names separated by a hyphen or a middle name initial when available)

Patient data will be securely stored for 10 years following trial completion.

**8.4 INDEMNITY**

North Bristol NHS Trust holds standard NHS Hospital Indemnity and insurance cover with NHS Litigation Authority for NHS Trusts in England, which apply to this trial.

**8.5 SPONSOR**

North Bristol NHS Trust will act as the Sponsor for this trial. Delegated responsibilities will be assigned to the NHS trusts taking part in this trial.

**8.6 FUNDING**

Academy of Medical Sciences. Underwritten by St George’s University Respiratory Research Fund.

**8.7 AUDITS**

The study may be subject to inspection and audit by North Bristol NHS Trust under their remit as sponsor and other regulatory bodies to ensure adherence to GCP and the NHS Research Governance Framework for Health and Social Care (2nd edition).

**9. STUDY MANAGEMENT**

The day-to-day management of the study will be co-ordinated through the Respiratory Research Unit, Southmead Hospital, BS10 5NB.

**10. PUBLICATION POLICY**

The study data will be published in peer review journals and presented at conferences and to local patient groups. Participants will not be identified in any publications.

**Summary of investigations, treatment and assessments**

Visit 1

|  | **Duration** |
| --- | --- |
| **Consent** | 20min |
| **Clinical Interview** | 10min |
| **Questionnaires** | |
| **Cognition (MoCA)**  **Health Status (CAT)**  **Mood (HADS)** | 10min  2min  5min |
| **Spirometry** | 5min |
| **Visual Acuity** | 3min |
| **Total** | **55min** |

Visit 2

|  | **Duration** |
| --- | --- |
| **Arterial Stiffness** | 10min |
| **Blood Test & blood gas measurement** | 10min |
| **Urine dipstick** | 2 min |
| **Total** | **22min** |

**Visit 3**

|  | **Duration** |
| --- | --- |
| **Safety Check** | 5min |
| **MRI brain** | 45min |
| **Break** | 10min |
| **MRI cardiac** | 20min |
| **Total** | **80min** |

**Visit 4**

|  | **Duration** |
| --- | --- |
| **Retinal Photography** | **20min** |

Acronyms:

MoCA - Montreal Cognitive Assessment

HADS - Hospital Anxiety and Depression Score

ABG - Arterial Blood Gas

**11. REFERENCES**

1. Rabe KF, Hurd S, Anzueto A, Barnes PJ, Buist SA, Calverley P, et al. Global strategy for the diagnosis, management, and prevention of chronic obstructive pulmonary disease: GOLD executive summary.[see comment]. [Review] [267 refs]. American Journal of Respiratory & Critical Care Medicine. 2007;176(6):532-55.

2. Agusti A. Chronic obstructive pulmonary disease: beyond the forced expiratory manoeuvre. Respiration. 2008;75(2):136-7.

3. Agusti AG, Noguera A, Sauleda J, Sala E, Pons J, Busquets X. Systemic effects of chronic obstructive pulmonary disease. [Review] [169 refs]. European Respiratory Journal. 2003;21(2):347-60.

4. Mannino DM, Buist AS. Global burden of COPD: risk factors, prevalence, and future trends. The Lancet. //;370(9589):765-73.

5. Dodd JW, Getov SV, Jones PW. Cognitive function in COPD. [Review] [94 refs]. European Respiratory Journal. 2010;35(4):913-22.

6. Dodd JW, Chung AW, van den Broek MD, Barrick TR, Charlton RA, Jones PW. Brain Structure and Function in Chronic Obstructive Pulmonary Disease. A Multi-Modal Cranial Magnetic Resonance Imaging Study. American Journal of Respiratory and Critical Care Medicine. 2012.

7. Lahousse L, Vernooij MW, Darweesh SKL, Akoudad S, Loth DW, Joos GF, et al. Chronic Obstructive Pulmonary Disease and Cerebral Microbleeds: The Rotterdam Study. American Journal of Respiratory and Critical Care Medicine. 2013.

8. Domino EF. Tobacco smoking and MRI/MRS brain abnormalities compared to nonsmokers. [Review] [67 refs]. Progress in Neuro-Psychopharmacology & Biological Psychiatry. 2008;32(8):1778-81.

9. Swan GE, Lessov-Schlaggar CN. The effects of tobacco smoke and nicotine on cognition and the brain. [Review] [162 refs]. Neuropsychology Review. 2007;17(3):259-73.

10. Longstreth WT, Jr., Manolio TA, Arnold A, Burke GL, Bryan N, Jungreis CA, et al. Clinical correlates of white matter findings on cranial magnetic resonance imaging of 3301 elderly people. The Cardiovascular Health Study. Stroke. 1996;27(8):1274-82.

11. Liao D, Higgins M, Bryan NR, Eigenbrodt ML, Chambless LE, Lamar V, et al. Lower pulmonary function and cerebral subclinical abnormalities detected by MRI: the Atherosclerosis Risk in Communities study. Chest. 1999;116(1):150-6.

12. Sachdev PS, Anstey KJ, Parslow RA, Wen W, Maller J, Kumar R, et al. Pulmonary function, cognitive impairment and brain atrophy in a middle-aged community sample. Dementia & Geriatric Cognitive Disorders. 2006;21(5-6):300-8.

13. Borson S, Scanlan J, Friedman S, Zuhr E, Fields J, Aylward E, et al. Modeling the impact of COPD on the brain. International Journal of Copd. 2008;3(3):429-34.

14. Rodriguez-Roisin R, Llufriu S. White and Gray Matter Impairment in Chronic Obstructive Pulmonary Disease. American Journal of Respiratory and Critical Care Medicine. 2012 2012/08/01;186(3):207-8.

15. Rodriguez-Roisin R, Llufriu S, Fabbri LM. Changes in Your Breathing Can Change Your Brain. American Journal of Respiratory and Critical Care Medicine. 2013 2013/10/01;188(7):763-4.

16. Maclay JD, McAllister DA, Macnee W. Cardiovascular risk in chronic obstructive pulmonary disease. Respirology. 2007;12(5):634-41. PubMed PMID: 17875049.

17. Feary JR, Rodrigues LC, Smith CJ, Hubbard RB, Gibson JE. Prevalence of major comorbidities in subjects with COPD and incidence of myocardial infarction and stroke: a comprehensive analysis using data from primary care. Thorax. 2010;65(11):956-62.

18. Anthonisen NR, Skeans MA, Wise RA, Manfreda J, Kanner RE, Connett JE. The Effects of a Smoking Cessation Intervention on 14.5-Year MortalityA Randomized Clinical Trial. Annals of internal medicine. 2005;142(4):233-9.

19. Mitchell GF. Effects of central arterial aging on the structure and function of the peripheral vasculature: implications for end-organ damage. Journal of Applied Physiology. 2008;105(5):1652-60.

20. Poels MlMF, Zaccai K, Verwoert GC, Vernooij MW, Hofman A, van der Lugt A, et al. Arterial Stiffness and Cerebral Small Vessel Disease: The Rotterdam Scan Study. Stroke. 2012;43(10):2637-42.

21. Sabit R, Bolton CE, Edwards PH, Pettit RJ, Evans WD, McEniery CM, et al. Arterial Stiffness and Osteoporosis in Chronic Obstructive Pulmonary Disease. American Journal of Respiratory and Critical Care Medicine. 2007;175(12):1259-65.

22. McAllister DA, Maclay JD, Mills NL, Mair G, Miller J, Anderson D, et al. Arterial Stiffness Is Independently Associated with Emphysema Severity in Patients with Chronic Obstructive Pulmonary Disease. American Journal of Respiratory and Critical Care Medicine. 2007 2007/12/15;176(12):1208-14.

23. Maclay JD, MacNee W. Cardiovascular disease in copd: Mechanisms. CHEST Journal. 2013;143(3):798-807.

24. Grant I, Heaton RK, McSweeny AJ, Adams KM, Timms RM. Neuropsychologic findings in hypoxemic chronic obstructive pulmonary disease. Archives of Internal Medicine. 1982;142(8):1470-6.

25. Antonelli I, Corsonello A, Pedone C, Trojano L, Acanfora D, Spada A, et al. Drawing impairment predicts mortality in severe COPD.[see comment]. Chest. 2006;130(6):1687-94.

26. Dodd JW, Charlton RA, van den Broek MD, Jones PW. Cognitive Dysfunction In Patients Hospitalized With Acute Exacerbation of COPD. Chest. 2013;Published online.

27. Dodd JW, Charlton RA, van den Broek MD, Jones PW. The Impact of Exacerbation on Cognitive Function in COPD. American Journal of Respiratory & Critical Care Medicine. 2012;185(A5855).

28. Curkendall SM, deLuise C, Jones JK, Lanes S, Stang MR, Goehring Jr E, et al. Cardiovascular Disease in Patients with Chronic Obstructive Pulmonary Disease, Saskatchewan Canada: Cardiovascular Disease in COPD Patients. Annals of Epidemiology. 2006 1//;16(1):63-70.

29. Mannino DM, Thorn D, Swensen A, Holguin F. Prevalence and outcomes of diabetes, hypertension and cardiovascular disease in COPD. European Respiratory Journal. 2008;32(4):962-9.

30. Fabbri LM, Beghé B, Agusti A. Cardiovascular mechanisms of death in severe COPD exacerbation: time to think and act beyond guidelines. Thorax. 2011 September 1, 2011;66(9):745-7.

31. Smith BM, Kawut SM, Bluemke DA, Basner RC, Gomes AS, Hoffman E, et al. Pulmonary hyperinflation and left ventricular mass: the Multi-Ethnic Study of Atherosclerosis COPD Study. Circulation. 2013;127(14):1503-11, 11e1-6. PubMed PMID: 23493320.

32. Murphy CA, Blyth KG, Chaudhuri R, Lafferty J, Hothersall E, Steedman T, et al. Assessment of the presence of occult myocardial infarction in chronic obstructive pulmonary disease using contrast-enhanced cardiac magnetic resonance imaging. Respiration. 2009;78(3):263-9. PubMed PMID: 19223680.

33. Vonk-Noordegraaf A, Marcus JT, Holverda S, Roseboom B, Postmus PE. EArly changes of cardiac structure and function in copd patients with mild hypoxemia*. CHEST Journal. 2005;127(6):1898-903.

34. Barr RG, Bluemke DA, Ahmed FS, Carr JJ, Enright PL, Hoffman EA, et al. Percent Emphysema, Airflow Obstruction, and Impaired Left Ventricular Filling. New England Journal of Medicine. 2010;362(3):217-27. PubMed PMID: 20089972.

35. Incalzi RA, Corsonello A, Pedone C, Battaglia S, Paglino G, Bellia V, et al. Chronic renal failure: a neglected comorbidity of COPD. Chest. 2010;137(4):831-7. PubMed PMID: 19903974.

36. Gjerde B, Bakke PS, Ueland T, Hardie JA, Eagan TML. The prevalence of undiagnosed renal failure in a cohort of COPD patients in western Norway. Respiratory Medicine. 2012;106(3):361-6. PubMed PMID: 22129490.

37. Karalliedde J, Viberti G. Microalbuminuria and cardiovascular risk. American journal of hypertension. 2004 Oct;17(10):986-93. PubMed PMID: 15485765. Epub 2004/10/16. eng.

38. Cooper LS, Wong TY, Klein R, Sharrett AR, Bryan RN, Hubbard LD, et al. Retinal Microvascular Abnormalities and MRI-Defined Subclinical Cerebral Infarction: The Atherosclerosis Risk in Communities Study. Stroke. 2006 January 1, 2006;37(1):82-6.

39. van den Heuvel MP, Hulshoff Pol HE. Exploring the brain network: a review on resting-state fMRI functional connectivity. [Review]. European Neuropsychopharmacology. 2010;20(8):519-34.

40. Zuo XN, Kelly C, Adelstein JS, Klein DF, Castellanos FX, Milham MP. Reliable intrinsic connectivity networks: test-retest evaluation using ICA and dual regression approach. Neuroimage. 2010;49(3):2163-77.

41. Smith SM, Jenkinson M, Johansen-Berg H, Rueckert D, Nichols TE, Mackay CE, et al. Tract-based spatial statistics: Voxelwise analysis of multi-subject diffusion data. Neuroimage. 2006;31(4):1487-505.

42. Smith SM, Nichols TE. Threshold-free cluster enhancement: Addressing problems of smoothing, threshold dependence and localisation in cluster inference. Neuroimage. 2009;44(1):83-98.

43. Smith SM. Fast robust automated brain extraction. [Review] [16 refs]. Human Brain Mapping. 2002;17(3):143-55.

44. Detre JA, Zhang W, Roberts DA, Silva AC, Williams DS, Grandis DJ, et al. Tissue specific perfusion imaging using arterial spin labeling. NMR in Biomedicine. 1994;7(1-2):75-82.

45. Petersen ET, Zimine I, Ho Y-CL, Golay X. Non-invasive measurement of perfusion: a critical review of arterial spin labelling techniques. British Journal of Radiology. 2006 August 1, 2006;79(944):688-701.

46. Barr RG, Mesia-Vela S, Austin JH, Basner RC, Keller BM, Reeves AP, et al. Impaired flow-mediated dilation is associated with low pulmonary function and emphysema in ex-smokers: the Emphysema and Cancer Action Project (EMCAP) Study. American Journal of Respiratory and Critical Care Medicine. 2007;176(12):1200.

47. Eickhoff P, Valipour A, Kiss D, Schreder M, Cekici L, Geyer K, et al. Determinants of Systemic Vascular Function in Patients with Stable Chronic Obstructive Pulmonary Disease. American Journal of Respiratory and Critical Care Medicine. 2008 2008/12/15;178(12):1211-8.

48. Pauca AL, O’Rourke MF, Kon ND. Prospective Evaluation of a Method for Estimating Ascending Aortic Pressure From the Radial Artery Pressure Waveform. Hypertension. 2001 October 1, 2001;38(4):932-7.

49. Yasmin, McEniery CM, Wallace S, Mackenzie IS, Cockcroft JR, Wilkinson IB. C-Reactive Protein Is Associated With Arterial Stiffness in Apparently Healthy Individuals. Arteriosclerosis, Thrombosis, and Vascular Biology. 2004 May 1, 2004;24(5):969-74.

50. Nasreddine ZS, Phillips NA, Bédirian V, Charbonneau S, Whitehead V, Collin I, et al. The Montreal Cognitive Assessment, MoCA: A Brief Screening Tool For Mild Cognitive Impairment. Journal of the American Geriatrics Society. 2005;53(4):695-9.

51. Jones PW, Harding G, Berry P, Wiklund I, Chen WH, Kline LN. Development and first validation of the COPD Assessment Test. European Respiratory Journal. 2009;34(3):648-54.

52. Zigmond AS, Snaith RP. The hospital anxiety and depression scale. Acta Psychiatrica Scandinavica. 1983;67(6):361-70.

53. Brusasco V, Crapo R, Viegi G. Coming together: the ATS/ERS consensus on clinical pulmonary function testing. European Respiratory Journal. 2005;26(1):1-2.
