# Supplementary figures and images for: Simple non-mydriatic retinal photography is feasible and demonstrates retinal microvascular dilation in Chronic Obstructive Pulmonary Disease (COPD)
Source: PLoS One. 2020 Jan 10;15(1):e0227175. doi: 10.1371/journal.pone.0227175 (PMC6953864; doi:10.1371/journal.pone.0227175)

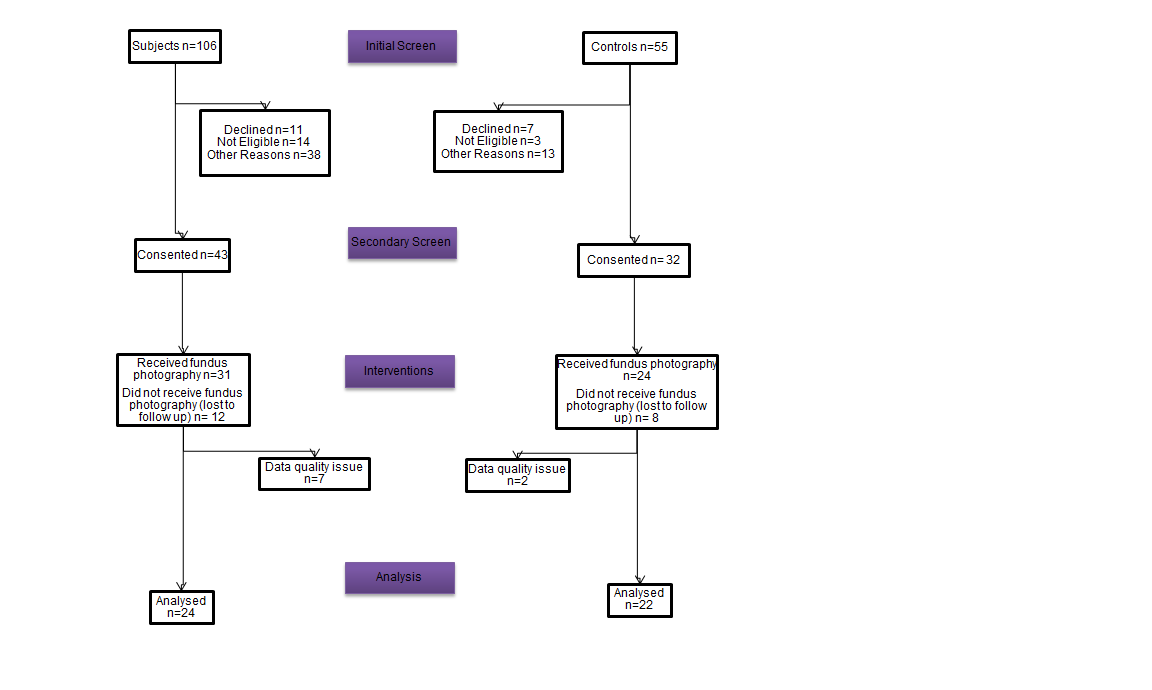

Supplement: S1 Flow Chart — (TIFF) [file pone.0227175.s003.tiff]
